# Horizon Scanning for Medical Technologies: Methodological Framework Development Study of the Medical Innovation Scanning Techniques (MIST) Framework

**DOI:** 10.2196/93166

**Published:** 2026-05-21

**Authors:** Sonia Garcia Gonzalez-Moral, Andrew Mkwashi, Fiona R Beyer, Dawn Craig

**Affiliations:** 1 NIHR Innovation Observatory at Population Health Sciences Institute, Newcastle University, The Catalyst Room 3.12, 3 Science Square Newcastle Helix, Newcastle upon Tyne, England, NE4 5TG, United Kingdom, 44 0191-2082262

**Keywords:** horizon scanning, evidence synthesis, information retrieval, methods framework, Medical Innovation Scanning Techniques

## Abstract

**Background:**

Strategic foresight relies on horizon scanning to detect weak signals of innovation and anticipate disruption in fast-moving MedTech. While tools such as the Rumsfeld matrix and the Three Horizons model structure thinking about uncertainty and time, their application in MedTech is limited and insufficient for addressing the regulatory, technical, and evidential complexity of emerging or disruptive technologies. Regulatory frameworks provide stability through defined safety and performance pathways, but shifts and regional variability requirements complicate anticipatory assessment. These challenges expose methodological gaps in current practice: broad foresight frameworks lack the technical granularity, regulatory alignment, and systematic processes required for MedTech, where rapid innovation intensifies uncertainty. This paper presents a structured methodological framework to support systematic, reproducible, and decision-focused horizon scanning in MedTech.

**Objective:**

The framework aims to (1) standardize methods for planning, execution, and reporting; (2) reduce uncertainty through systematic identification and interpretation of weak signals; and (3) improve transparency, quality, and comparability across policy, regulatory, clinical, and strategic contexts.

**Methods:**

The framework was developed following the 3-step process by McMeekin et al. First, data were identified through a mapping review of MedTech futures and foresight methods, mapping UK health care decision-makers, and participating in national and international horizon scanning initiatives (2021‐2025). Second, the framework was constructed by integrating the sources, methods, and insights with the principles of regulatory and technology readiness, supported by ongoing consultation with UK regulatory and health system bodies. Third, the framework is being applied and tested in MedTech horizon scanning projects. Formal validation and iterative revision are planned.

**Results:**

The MedTech Innovation Scanning Techniques (MIST) framework integrates foresight theory, horizon scanning methods, and the MedTech innovation pathway, enabling systematic weak signal detection across 3 time horizons. In the imminent horizon (technology readiness level [TRL] 8‐9), uncertainty is lowest, and weak signals are more visible, supported by rapid evidence synthesis and expert engagement. In the transitional horizon (TRL 4‐7), uncertainty increases due to trial outcomes, funding, and market dynamics, requiring broader evidence sources and complementary techniques such as scenario analysis and bibliometrics. In the emerging horizon (TRL 1‐3), weak signals are abundant but least predictable; early-stage data (patents, preclinical research, and conference outputs) are analyzed using methods such as term-similarity visualization to reveal nascent innovations. Across all horizons, MIST supports systematic signal identification, contextualization, and prioritization to inform anticipatory decision-making.

**Conclusions:**

MedTech horizon scanning is challenging. MIST addresses these challenges by integrating the Three Horizons model, Rumsfeld matrix, technology pathways, regulatory considerations, and sources to guide weak signal detection. MIST provides a structured, transparent, and reproducible approach tailored to MedTech. While formal validation and integration of equity and ethical dimensions are ongoing, the framework fills a critical methodological gap in horizon scanning for MedTech.

## Introduction

### Overview

Strategic foresight is often referred to as “futures studies” or “futures research” and is a discipline used by organizations to gather and process information about their future operating environment. This may encompass trends and developments that shape the organization’s political, economic, social, technological, legal, and environmental context [1].

Horizon scanning, referred to as the “the systematic identification of new and emerging health technologies that have the potential to impact health, health services, and/or society” [2], is a crucial tool for health and care decision-making, enabling health and care systems to anticipate and adapt to rapid technological change; providing policy decision-makers with high-quality, early intelligence on emerging health technologies, to enable them to navigate pressures from innovation demands and constrained resources; while also allowing the wider health and life science sector stakeholders to use the same intelligence to drive innovation in areas of unmet need for the benefit of patients and the economy. As a strategic foresight tool, horizon scanning systematically detects weak signals of technology emergence and development, providing early insights that support system and sector preparedness. This proactive approach not only identifies opportunities for market growth and innovation but also mitigates political and operational risks, giving policymakers the time and evidence needed for informed planning and resilient health systems [3,4].

MedTech refers to the broad spectrum of health services, products, and solutions (classified as medical devices, in vitro diagnostics, or digital health applications) designed to enhance health outcomes and health and care delivery. These technologies play a crucial role in transforming care pathways, improving health, and generating cost efficiencies [5]. MedTech innovations have redefined health care delivery through improved monitoring, accessibility, and diagnostic precision. For example, wearable health monitors such as continuous glucose monitors exemplify how continuous physiological tracking can enhance preventive and chronic disease management. Artificial intelligence (AI)–powered diagnostic tools have revolutionized clinical decision-making by automating disease detection, and robotic surgery systems have enhanced surgical precision and minimized invasiveness. The MedTech field is marked by rapid innovation, with products typically undergoing short lifecycles (18‐24 months), with frequent updates or entirely new innovations emerging [5]. The development of medical devices involves a complex, iterative, and interdisciplinary process, from needs identification to conceptualization and final product realization [6], engaging clinicians, engineers, business professionals, and patients along the way.

Despite common agreement on horizon scanning as a tool for signal detection, anticipatory innovation decision-making, implementation, dissemination, and unmet needs identification [7] in health care, the heterogeneous application of horizon scanning and foresight methods, coupled with inconsistent reporting and the absence of best practice consensus, hampers the quality and timeliness of MedTech innovation assessments. Furthermore, a recently published mapping review identified that the term “horizon scanning” was inconsistently applied and vaguely defined in the literature, often encompassing a broad mix of data sources and stakeholder consultations [8]. Although many definitions exist, most support the concept that horizon scanning is a method that aims to systematically detect signals of change or “weak signals” which may be understood as early signs of emerging technologies.

### The Current Unmet Methodological Need

Horizon scanning in health technology, especially MedTech, faces unique methodological, practical, and conceptual challenges that make the case for providing structured practical guidance and specialized tools to guide users through each step of the process. Below, we discuss this need, the role of standardization and systematization, and why established foresight frameworks (such as Framework Foresight [9]) are not fully translatable to MedTech horizon scanning.

First, MedTech involves fast, iterative innovation, diverse product types with varied evidence needs, complex multistakeholder ecosystems, and safety risks tied to user interaction and system integration. These features require practical, structured Horizon scanning tools that ensure transparency, reproducibility, and context-specific applicability [8,10].

Second, established foresight frameworks such as Framework Foresight [9,11,12] or the European Commission Concurrent Design Foresight [13] offer broad futures-thinking structures but treat horizon scanning as an early, general step rather than a detailed, stand-alone method. They lack the technical, regulatory, and prioritization guidance needed for MedTech, serving more as conceptual guides than practical, MedTech-specific tools.

Lastly, in MedTech, where evidence gaps and complex trends heighten uncertainty, standardized and systematic horizon scanning methods are vital. They promote transparency, consistency, and reproducibility; enable auditable signal detection and prioritization; and use structured tools to reduce bias and missed developments. Literature shows this approach builds trust, supports benchmarking, and fosters shared learning [14].

### Aims and Objectives

This paper aims to provide a transparent and detailed account of the development of a structured methodological framework to guide the application of horizon scanning in the context of emerging MedTech innovations. The framework is intended to offer clear, practical steps that support systematic, reproducible, and decision-oriented horizon scanning practice.

### Objectives

The development of this framework was guided by three core objectives which were (1) to standardize horizon scanning methods by defining essential methodological components and offering a coherent structure that supports consistent planning, execution, and reporting of horizon scanning activities; (2) to reduce uncertainty surrounding new and emerging MedTech innovations through systematic identification, assessment, and interpretation of early signs and future developments relevant to health and care; and (3) to strengthen evidence-informed decision-making by improving the transparency, quality, and comparability of horizon scanning outputs used to inform policy, regulatory, clinical, and strategic decisions regarding MedTech innovation.

## Methods

### Study Design

For the creation of the framework, we followed the steps outlined by McMeekin et al [15] that consisted of (1) identifying the data to inform the methodological framework, (2) developing the framework, and (3) testing and validating this framework in case studies, and refining the framework.

We planned to add an additional step, step 4: revision and refining of the framework. Although this last step is considered by McMeekin et al [15] as part of step 3, given the foreseen impact that changes introduced to the framework may have in practice and the number of potential framework users affected by them, a systematic and transparent approach is essential to ensure credibility and usability whereby all additional change sustained after the publication of the first iteration of the framework will be disseminated separately providing a rationale for the change and a comparative analysis.

### Step One: “Identifying the Data”

This step was undertaken with a mapping review of futures and foresight methods used for the identification of emerging and innovative MedTech [8]. This review identified different sources and purposes for the practice of horizon scanning in the context of MedTech and the main range of stakeholders involved in the process. These provided the epistemological foundation of the framework. Additionally, to make this framework more responsive to UK horizon scanning practice, we mapped the characteristics of all national health care decision-makers across the UK infrastructure [16]. A total of 39 structural bodies with decision-making capacity in health care were identified, as well as their interbody relationship within the system. This stakeholder mapping supported the identification of priority areas and gaps within the system and provided the basis for the design of proactive horizon scanning. Alongside these 2 major projects, between 2021 and 2025, members of this team (SGG-M and AM) participated in several national horizon scanning initiatives (Table 1).

**Table 1.** Horizon scanning initiatives.

Name of initiative	Purpose	Dates	Attendee
AAC^a^ horizon scanning working group	Devise methods for horizon scanning innovative health care technologies	2020‐2022	SGG-M
Medicines and Medical Devices Initiative Medical Devices Working Group	Coordinate different stakeholder needs to streamline horizon scanning practice and increase efficiency across the system	2022‐2025 2023-2025	SGG-M AM
Nuffield Council on Bioethics	Identify opportunities for embedding ethics in the horizon scanning of new technologies	2023‐2025	SGG-M
International Horizon Scanning Initiative	Establish a horizon scanning service and terms of reference for standardizing the practice of horizon scanning for MedTech from different countries’ economic perspectives, needs, and regulatory frameworks	2022‐2025	SGG-M
International HealthTechScan	Promotes early awareness via horizon scanning practice	2020‐2025 2023‐2025	SGG-M AM

a AAC: Accelerated Access Collaborative.

### Step Two: “Developing the Methodological Framework”

This step encompassed several existing methods, theories, sources, and regulations that mitigate the uncertainty of the unknowns. This was mainly done by integrating various horizon scanning methods outlined in the literature [17], innovation frameworks [18], sources identified from horizon scanning practice and from the literature [8,17,19], working group insights, and relevant publications [10], professional experience with established technology readiness levels (TRLs) [20,21], and regulatory frameworks [22-25] to understand how to work with uncertainty [1,26]. The regulatory aspect of the framework is a constantly evolving element as regulators respond to innovation. To understand the latest challenges and initiatives, informational consultation with national stakeholders, including the Medicines and Healthcare Products Regulatory Agency, Healthcare Improvement Scotland, and Health Technology Wales, was undertaken at regular intervals. This step was undertaken iteratively, being informed by the constant change in the regulatory landscape and ad hoc, during “contact points” with regulatory experts via online meetings.

### Step Three: “Testing and Validating the Methodological Framework”

This step is ongoing as one of the core foundational commitments of the Innovation Observatory (IO)—the UK national horizon scanning center—to methods development in this field. The authors, in their capacity as researchers, are taking an agile approach to testing and validating this methodological framework implicit to all IO’s MedTech horizon scanning projects. The application of this framework starts with the scoping of the topic, the establishment of the horizon, sources, and TRLs, as well as the analysis and reporting of the data. A formal validation of this framework is expected in the near future.

### Ethical Considerations

This study did not involve human participants, patient data, or identifiable personal information. The work consisted solely of methodological development activities conducted by professionals in their occupational roles and analysis of publicly available institutional reports. According to institutional and national research governance guidance, this type of study does not constitute human subjects research and therefore did not require review or approval by an institutional review board or research ethics committee [27]. All procedures were conducted in accordance with applicable institutional, national, and international standards for research integrity and publication ethics [28].

## Results

### Step 1: Identifying the Data

#### Main Sources of Weak Signal Detection

Through our previously published research [8] and expert horizon scanning practice, we have identified the breadth and relative frequency of information sources used in horizon scanning activities (Table 2). Specialist journals and medical bibliographic databases dominate, reflecting the continued centrality of the academic literature. These are followed by patent registries, expert input, and outputs from other horizon-scanning agencies, highlighting the importance of early-stage innovation intelligence and peer networks. Clinical trial registries and industry, funders, or regulatory sources occupy a midtier, supporting pipeline visibility and regulatory awareness. A wide array of supplementary sources, such as commercial research and development databases, web searches, news outlets, social media, and conference outputs, are used less frequently but contribute to signal detection and contextual awareness. Collectively, the distribution and heterogeneity of sources demonstrate that effective horizon scanning relies on triangulating formal scientific evidence with regulatory, funding, commercial, and informal intelligence streams to capture emerging technologies at different stages of maturity.

**Table 2.** Sources used in MedTech horizon scanning.

Category and name	Content	Access
Bibliographic databases		
IEEE Xplore	Engineering	Free
EMBASE (Ovid)	Medical	Paywall
Engineering Village (Elsevier)	Engineering	Paywall
Specialist publications		Free
Medical device network	MedTech news, regulatory news, and company intelligence.	
Med-tech news	MedTech innovations, market intelligence, companies’ news, and health and social care policy news.	
Medical technology outlook	MedTech news, conferences, and insights.	
Today’s medical developments	Industry news, manufacturers, components and materials, medical technology innovators, and regulatory decisions.	
Clinical trial registers		Free
ClinicalTrials.gov	Clinical trial protocols of ongoing and completed research. US and non-US trials.	
EUdraCT; CTIS^b^	EUdraCT: Clinical trial protocols of ongoing and completed studies undertaken in any EU^a^ country up to January 2022. CTIS: From January 2022.	
CTIS	New European clinical trial platform for all ongoing clinical trials in the EU. Supersedes the previous clinical trial portal (included above)	
WHO^c^ ICTRP^d^ Search Portal	ICTRP from the WHO. International scope.	
ScanMedicine	A one-stop shop for global clinical trial studies ongoing or completed, registered in more than 11 different national registers, including the US and the European Union. Developed by the NIHR Innovation Observatory, this source is free.	
Regulatory databases		
FDA^e^ Medical Devices	PMA^f^, 510(k) premarket notifications and De Novo	Free
Patent databases		
Lens.org	Over 150 million patent records with a database that is updated every 2 weeks. International patent information is available from 106 jurisdictions.	Free^g^
PATENTSCOPE	PATENTSCOPE is a free patent database made available by the WIPO^h^ with coverage of over 60 patent collections. It is the primary and thus most up-to-date and complete source of data on patent applications filed under the PCT^i^. It contains 115.7 million patent documents.	Free
News		
Google News (Google LLC)	News aggregator by Google LLC. It provides access to thousands of news outlets and is constantly updated.	Free
Funding and grants		
Various sources	Funding calls (open and closed) Grants and principal investigators	Free

a EU: European Union.

b CTIS: Clinical Trials Information System.

c WHO: World Health Organization.

d ICTRP: International Clinical Trials Registry Platform.

e FDA: US Food and Drug Administration.

f PMA: premarket approvals.

g Open Public Access for individuals wanting to search and analyze patents and scholarly works. Access for organizations or to additional resources is subscription-only.

h WIPO: World Intellectual Property Organization.

i PCT: Patent Cooperation Treaty.

#### The UK Stakeholder Map

**Figure 1.**
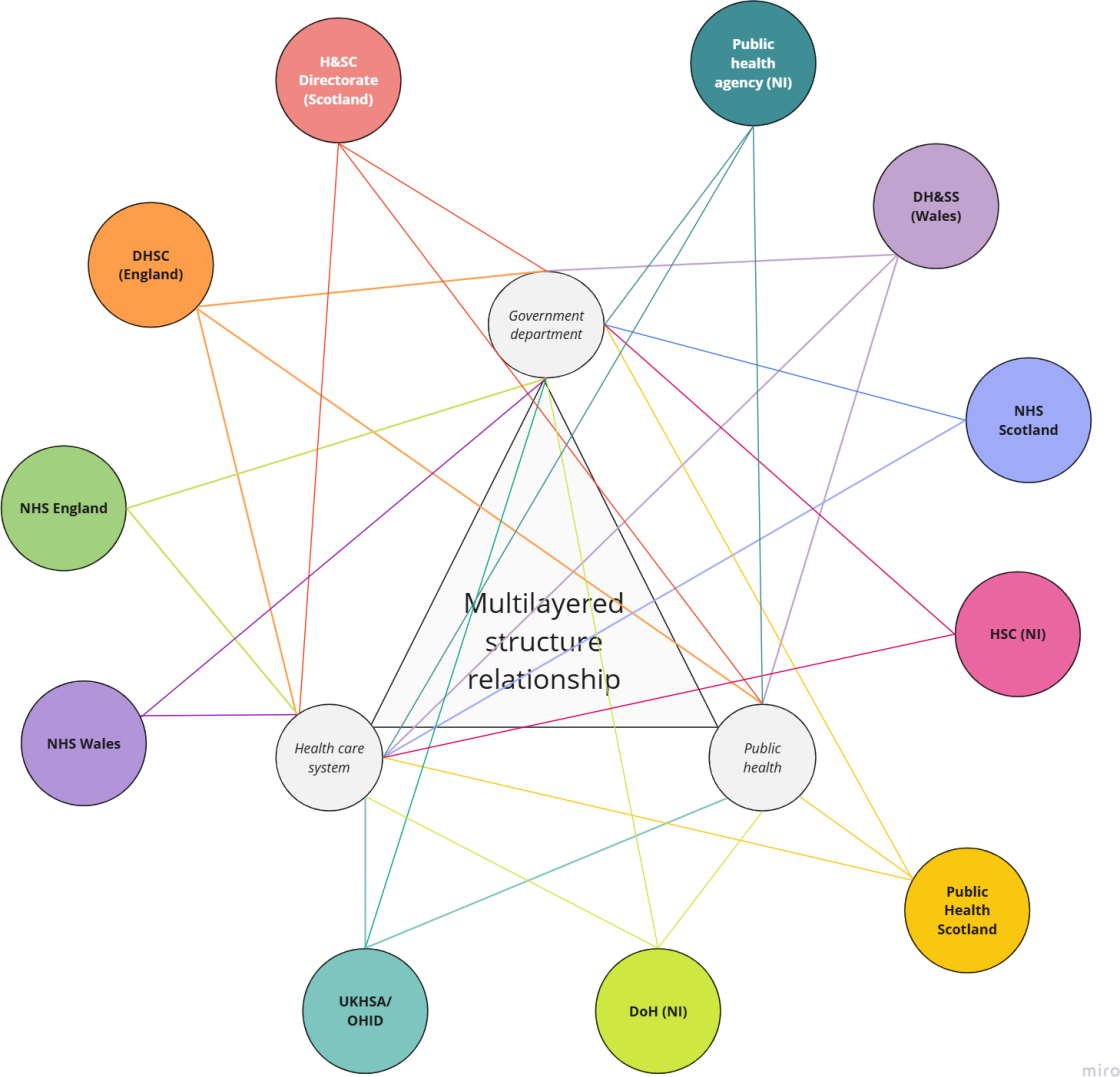
UK stakeholders map. DH&SS: Department for Health and Social Services; DHSC: Department of Health and Social Care; DoH: Department of Health; H&SC: Health and Social Care; HSC: Health and Social Care; NHS: National Health Service; NI: Northern Ireland; OHID: Office for Health Improvement and Disparities; UKHSA: United Kingdom Health Security Agency.

The UK health care decision-making landscape is characterized by a complex network of organizations, spanning mandatory decision-makers and advisory bodies, many of which engage directly with emerging MedTech innovations. In 2023, we mapped this ecosystem through iterative and snowballing searches, identifying 32 heterogeneous structures grouped into 7 functional categories (Figure 1): certification and health technology assessments (HTAs), advisory or leadership, clinical guidelines, health delivery, regulation and licensing, innovation accelerators, and access pathways. Several entities operate across multiple categories, highlighting the intricacy and interdependence of decision-making processes governing the adoption, implementation, and diffusion of new technologies [16]. Within this multiactor environment, horizon scanning functions as a cross-cutting intelligence layer; the 10 applications included in Table 3 have been extracted from published literature [8] and align closely with the distinct roles of these stakeholder groups.

**Table 3.** Application and use of horizon scanning by UK stakeholders.

Horizon-scanning purpose	Primary UK stakeholder groups served	Role in decision-making
Early warning	Advisory and leadership bodies; regulators; innovation accelerators	Anticipate disruptive or high-impact technologies; prepare policy, regulatory, and system responses
Identify emerging trends	Advisory and leadership; HTA^a^ bodies; innovation accelerators	Sense weak signals and trajectory shifts across MedTech domains
Identify future developments	HTA; clinical guideline developers; health delivery organizations	Inform pipeline awareness and forward planning
Support decision-making	HTA agencies; advisory bodies; commissioners	Provide structured intelligence to underpin formal recommendations
Technology assessment	HTA bodies; regulators	Frame technologies for evaluative processes and readiness appraisal
Planning	Health delivery; pathway and access bodies; NHS system planners	Enable workforce, infrastructure, and service planning
Predict impact in health care services	Health delivery; commissioners; advisory bodies	Anticipate system-level, clinical, and resource implications
Identify evidence gaps	HTA; research funders; guideline developers	Shape research agendas and reduce uncertainty
Adoption and diffusion	Pathway and access bodies; health delivery organizations	Accelerate appropriate uptake and scale-up
Identify business opportunities	Innovation accelerators; industry-facing bodies	Support innovation ecosystems and investment decisions

a HTA: health technology assessment.

Together, these findings provide the empirical backbone for the MedTech Innovation Scanning Techniques (MIST) framework by demonstrating that effective MedTech horizon scanning is inherently multisource, multistage, and stakeholder-contingent. The observed stratification of information sources (from specialist journals and bibliographic databases, through patents, trials, and regulatory data, to informal and gray channels) maps directly onto different phases of technological maturity and readiness. MIST operationalizes this by structuring scanning activities across foresight stages and technology-readiness levels, specifying which sources are most informative at each stage and why. The UK stakeholder mapping further grounds MIST in real-world decision contexts: the 10 horizon-scanning applications align with distinct functional roles across HTA, regulation, service delivery, and innovation acceleration. MIST provides a coherent methodological architecture that brings together interdependent methods, techniques, tools, and instruments, used systematically toward a common purpose and across multiple applications. It reflects horizon scanning not as a single method or tool, but as a configurable intelligence system, and translates that complexity into a structured, purpose-driven, and reproducible approach to MedTech horizon scanning by linking source typologies, innovation maturity, and the anticipatory needs of heterogeneous decision-makers.

### Step 2: Developing the Framework

#### Horizon Scanning Methods

Beyond the core methods such as expert panels, patent analysis, literature searching, stakeholder engagement, horizon scanning, and road mapping already identified [8,19], studies variably used Delphi, trend analysis, patient engagement, and text mining, alongside a long tail of infrequently used approaches, including clinical trial analysis, environmental scanning, projective scenarios, overlay mapping, and strategic foresight [8].

Across all studies, horizon scanning itself is the most frequently reported method, followed by expert consultation, literature searching, and patent analysis.

#### Methods for Working With Uncertainty

Horizon scanning encompasses weak signals of varying strength. Weak signals represent early indications of potential future developments and are typically characterized by a high degree of uncertainty. It is not always clear whether such weak signals are reliable, as errors in detection or data quality may lead to false positives. Moreover, even when genuine, the trajectory and potential impact of the anticipated change often remain uncertain at this early stage [29]. Acknowledging the uncertainty that surrounds weak signals is an important milestone for horizon scanning practitioners and commissioners. Ultimately, horizon scanning should not only detect the weak signals but also provide sufficient contextual information to help unpick the uncertainty that surrounds them. According to Garvey et al [1], “uncertainty is often less about the unknown as about the identification and interpretation of objectively known but hidden data and weak signals.”

There are several tools and techniques for supporting decision-making under uncertain conditions [30]. Among those, the Rumsfeld matrix has been used to characterize 4 different types of uncertainty (Figure 1).

According to this matrix, events are assessed according to their predictability or unpredictability and visibility or invisibility. The combination of those two-by-two options provides 4 different quadrants in which events may be classified and studied. The application of the Rumsfeld matrix to health care technology discovery may be a relatively unexplored area of research, but some studies are starting to use this tool to explore and categorize what is known and unknown to select pathways for investigation [31]. While the Rumsfeld matrix helps demystify uncertainty by classifying different types at play and proposes a framework to deal with each type, it fails to quantify the uncertainty and does not fully resolve the time variable. Timeliness impacts most HTAs and health care commissioning decision-making [32]; therefore, unpicking the time variable becomes crucial for effective horizon scanning methods.

#### Methods for Working With Time

**Figure 2.**
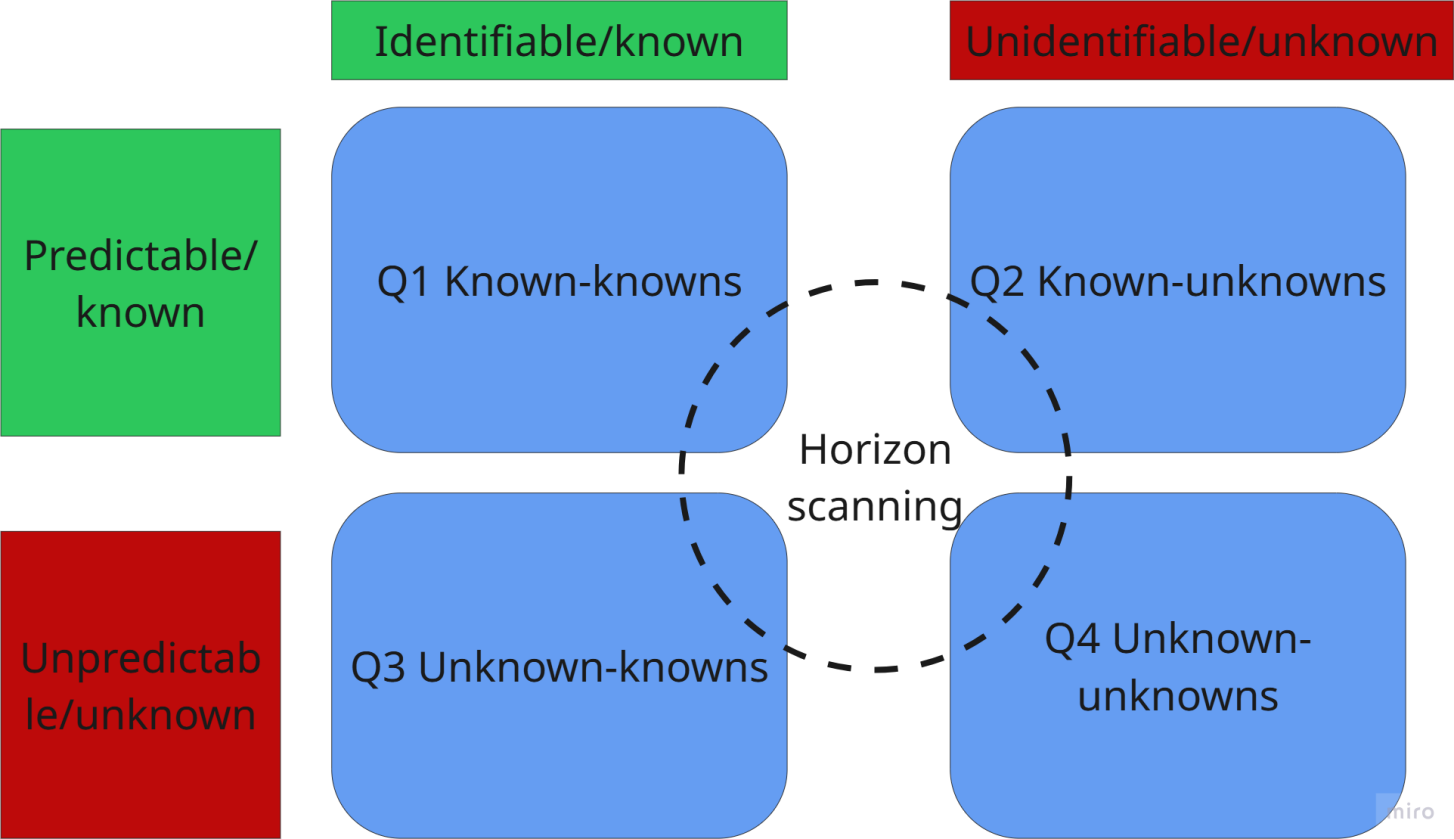
Rumsfeld matrix, predictability, and visibility profiles, and horizon scanning.

The Three Horizons model illustrates how issues, opportunities, and weak signals emerge across 3 distinct but interacting timeframes. Developed in the late 1990s with a focus on strategic management, the model encourages decision-makers to engage simultaneously with short-, medium-, and long-term futures [18,33,34]. In Figure 2, this model is applied specifically to MedTech technologies.

**Figure 3.**
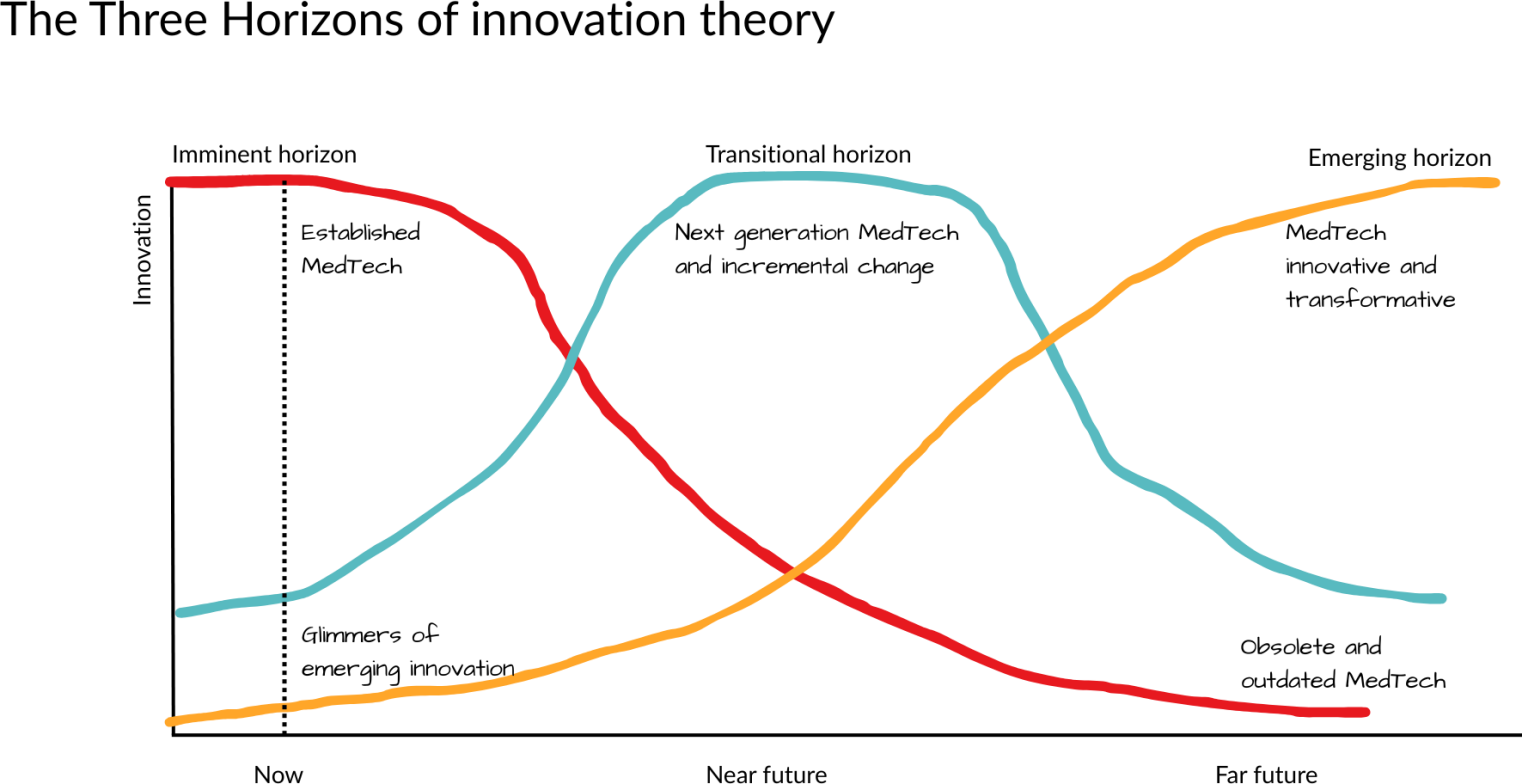
The horizons of innovation theory applied to MedTech.

Following the rationale exposed by Curry and Hodgson [33], the horizons are interpreted and presented in the following order (Figure 3):

Horizon 1 (H1, now) or “imminent horizon” represents the current state or “business as usual” and is associated with the present or near-term. It acknowledges that ongoing changes in the external environment will eventually disrupt this status quo, for example, established MedTech, diminishing its relevance over time (obsolescence) [33,35].

Horizon 3 (H3, far future) or “emerging horizon” reflects the long-term future, where new and uncertain developments begin to take shape; these may eventually replace or transform the current systems in H1. Although the specific nature of these changes is uncertain, monitoring them is essential, for they may reveal innovative and transformative technology that has the potential to radically change established health care pathways [33,36].

Horizon 2 (H2, near future) or “transitional horizon” serves as the transitional space between the present and the future, where emerging innovations and adaptive responses such as better iterations of already established health care products are tested in anticipation of broader shifts from H3 to H1 [33,37].

While this model provides a practical framework for working with time for innovation adoption and implementation, it does not solve the uncertainty that surrounds some of these emerging innovations. The key challenge is predicting how, when, and where new events or trends will occur and the level of disruption that these new and emerging innovations may generate. As these challenges involve many variables across multiple conditions or dimensions, additional surveillance or forecasting tools are needed to better understand the chance of an event occurring as well as the impact or level of disruption [38]. The Three Horizons model aims to alleviate uncertainties in the time dimension, but its capabilities are limited for other dimensions or technology disruptiveness levels.

#### The Pillars of Certainty: Regulatory Frameworks for MedTech

##### The Importance of Regulatory Frameworks

Regulatory frameworks are essential for ensuring the safety, efficacy, and quality of MedTech [39]. They provide a structured pathway for the development, approval, and postmarket surveillance of medical devices, in vitro diagnostics, and digital health applications [23,25]. These frameworks are particularly critical in MedTech due to the rapid pace of innovation, short product lifecycles, and the interdisciplinary nature of the field, which involves collaboration among clinicians, engineers, and regulatory experts [39].

##### The Role of Regulatory Frameworks in Reducing Uncertainty

Regulatory frameworks play a pivotal role in reducing uncertainty about a product’s intended use and safety requirements by establishing clear guidelines and standards for MedTech innovation. They help ensure that emerging technologies meet predefined safety and performance criteria, thereby mitigating risks associated with new products [40]. By providing a predictable approval process, these frameworks enable developers to anticipate challenges and align their strategies with regulatory expectations, ultimately fostering greater confidence in the adoption of new technologies [39]. Regulatory frameworks also contribute to reducing uncertainty in horizon scanning by providing a structured approach to evaluating the safety and efficacy of emerging technologies. For example, the integration of regulatory milestones, such as clinical trial phases and market authorization, into horizon scanning processes allows stakeholders to anticipate the trajectory of new technologies and their potential impact on health care systems [10].

##### Regulatory Challenges in MedTech

One of the key challenges in MedTech regulation is the variability across regions. For instance, in the European Union, the Medical Devices Regulation (MDR) and In Vitro Diagnostic Medical Devices Regulation impose stringent requirements for CE (Conformité Européenne) marking, while in the United Kingdom, post-Brexit regulations require UKCA (UK conformity assessment) marking for market access [41]. In the United States, the US Food and Drug Administration’s (FDA) Center for Devices and Radiological Health oversees device regulation [23]. These differing requirements can create complexity for developers seeking global market entry.

##### How Regulatory Changes Might Impact Horizon Scanning

Regulatory changes, such as the introduction of the European Union MDR and In Vitro Diagnostic Medical Devices Regulation, have significant implications for horizon scanning. These regulations require more rigorous postmarket surveillance and clinical evidence, which can affect the timeline and visibility of emerging technologies (European Union MDR, 2017). Horizon scanning practitioners must adapt to these changes by incorporating regulatory compliance data into their analyses, ensuring that the identified weak signals are not only innovative but also viable within the regulatory landscape [10]. Conversely, horizon scanning may signal new and innovative technology aspects not covered by current regulation, making this method a strategic tool for regulators [42,43].

##### The Novelty of Integrating the Three Horizons Model With MedTech Regulatory Frameworks

The integration of the Three Horizons model with MedTech regulatory frameworks represents a novel approach to managing uncertainty in horizon scanning. By aligning the model’s focus on short-, medium-, and long-term horizons with the staged regulatory pathways for MedTech development, this approach provides a comprehensive framework for tracking the progression of technologies from early innovation (horizon 3) to market readiness (horizon 1). This integration enhances the ability of horizon scanning practitioners to anticipate regulatory challenges and opportunities, thereby improving decision-making and preparedness for the adoption of disruptive technologies.

**Figure 4.**
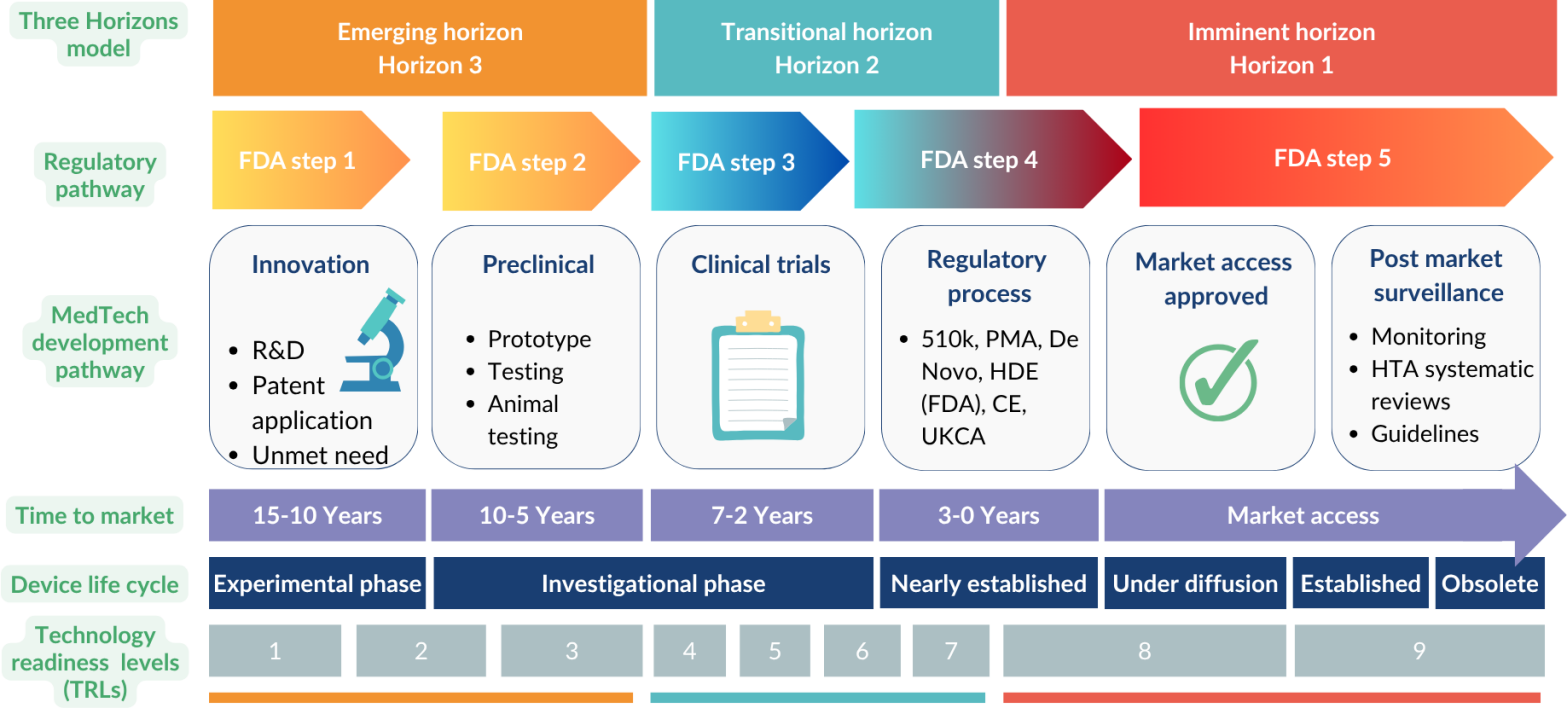
MIST MedTech horizon scanning framework: TRLs, FDA regulatory milestones, and horizons. CE: Conformité Européenne; FDA: US Food and Drug Administration; HDE: humanitarian device exemption; HTA: health technology assessment; PMA: premarket approval; R&D: research and development; TRL: technology readiness level; UKCA: UK conformity assessment.

Figure 4 presents the 3 horizons, TRLs in relation to their corresponding lifecycle stages, and predicted time to market according to the FDA regulatory pathway.

### Horizon Scanning in the Imminent Horizon (Horizon 1)

#### Overview

In this context, we understand the imminent horizon as the time between preregulatory processes and postmarket surveillance. The scope of this horizon is for technologies at a late stage of development (TRLs 8‐9) that are approaching their regulatory submission, undergoing regulatory certification and approval by a certified body, and the postmarket surveillance and monitoring activities for safety and effectiveness. The time in this horizon is short (less than 3 years to market access plus a variable amount of time for postmarket surveillance). This horizon overlaps with the HTA domain and systematic reviews of effectiveness due to evidence of the technologies’ effect and cost becoming available as technologies undergo the fundamental milestones of approval and market launch.

The inclusion of this horizon within MIST is important because horizon scanning is concerned not only with distant or highly uncertain futures, but also with anticipating near-term changes that may disrupt or rapidly reshape current systems. In MedTech, technologies in horizon 1 may be close to adoption, yet important uncertainties remain regarding regulatory progression, implementation, uptake, system readiness, and the displacement of existing practices. This horizon is also critical for identifying abrupt discontinuities, as understanding their immediate effects on the health care system is essential to support a timely response, preparedness, and consideration of alternative options should a sudden disruption occur. Including this horizon ensures that MIST captures the full continuum of innovation emergence, from early weak signals to technologies approaching real-world impact.

**Figure 5.**
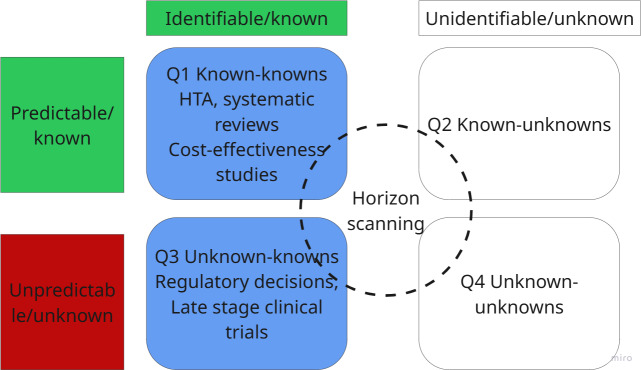
Rumsfeld matrix quadrants in the imminent horizon. HTA: health technology assessment.

In this horizon, the level of uncertainty about the future of a technology’s emergence is minimal, but the uncertainty of the technology’s effectiveness and cost-effectiveness is not resolved. This horizon corresponds to the known unknowns (Q3) and the known knowns (Q1) quadrants in the Rumsfeld matrix (Figure 5) [1].

In this horizon, the level of predictability of an event, or in this context, of a new technology occurring or emerging into the market, is potentially at the highest point in the technology development pathway (Q1). However, in Q3, unforeseen or invisible circumstances can obstruct the technology’s emergence, such as the lack of approval due to lack of compliance with regulations; a negative recommendation from a HTA body due to surpassing cost-effectiveness thresholds; or lack of technology adoption due to inequitable design aspects that interact with the effectiveness of the technology, for example, absence of skin color inclusivity for a technology whose mechanism of action is reading skin biomarkers [44].

The ability for horizon scanning practitioners to see these events ahead of time relies on the acquisition of in-depth detail about such technologies, as well as a deep understanding of the technology’s regulatory, political, societal, environmental, and economic dimensions. Textbox 1 introduces prompts for dimensional factors that could help horizon scanning practitioners improve the visibility of unforeseen events in this horizon.

Textbox 1. **Dimensional factors relevant for the imminent horizon.**

- Regarding the regulatory dimension, these may include risk class considerations, different regulatory pathways, the existence of clinical trials, and the intended use of the technology.
- Regarding the political dimension, are there policies that prioritize these technologies? Are national or international funding bodies backing up the development of these technologies?
- Regarding the societal dimension, will this technology provide a better experience of health and well-being to the entire society? Are they inclusive and designed with patients in mind? Do they address known unmet needs?
- Regarding the environmental dimension, are they sustainable or green? Will there be further iterations of these technologies, and how will they be implemented sustainably?
- Regarding the economic dimension, are these new technologies more cost-effective than existing ones? Will they trigger economic implications for patients and users? Are these technologies displacing obsolete ones? If so, are there measures in place for disinvesting in obsolete technology while we sustain growth and innovation?

#### Best Fit Methods, Sources, and Tools for Horizon Scanning in the Imminent Horizon

Table 4 presents a summary of methods that can be used by the many, including the IO, to identify and contextualize weak signals in the imminent horizon. These methods may be used alone or in combination, depending on variables such as the time frame available to deliver the project, the resources and capacity at hand, and the scope of the project. Most horizon scanning projects in the imminent horizon will commence with a rapid search for evidence. Rapid evidence synthesis methods provide a quick overview of a technological field by searching the published literature [45]. These methods can help identify weak signals that can serve as “leads” for discovering new weak signals, or they can provide contextual insights into the current state of research and innovation development within the relevant field. The findings of the review can guide subsequent decisions about where to search next and what to search for. Horizon scanning searches are characterized by sensitivity-maximizing searches across multiple sources. These searches aim to generate a vast amount of data, which is then screened and selected according to the scope of the horizon scan. This process may uncover new weak signals or “duplicate” ones that will strengthen the weak signals already identified via rapid evidence synthesis. While engagement with experts, patients, and the public might not always be required, these methods often yield rich qualitative data providing valuable contextual insights for assessing the dimensional factors in Textbox 1.

**Table 4.** Mapping of methods and sources for the imminent horizon.

Method used	Recommended sources
Rapid evidence synthesis	Published literature
Horizon scanning searches	Manufacturer sites Phase III and phase IV ongoing clinical trials Regulatory agencies Market scan Specialist MedTech publications
Sentiment analysis	Social media
Surveys and questionnaires	Patient and public engagement
Clinical and nonclinical topic expert workshops	Expert consultation

### Horizon Scanning in the Transitional Horizon (Horizon 2)

#### Overview

The transitional horizon refers to the period from the start of human clinical trials (pilot stage) to the initiation of preregulatory processes, typically during pivotal stage trials. This horizon focuses on technologies at mid-stage of development (TRLs 4‐7) that are commencing their investigational trials, generating evidence of their safety, efficacy, and outcomes, and compiling that evidence for certification. The duration of this horizon can vary significantly, heavily influenced by factors such as clinical investigation quality [46], market drivers [47], policy priorities [48], or investor support, among others. For low-risk devices, clinical trials might not be required, making this stage shorter than for high-risk devices [25]. Typically, this stage can last between 2 and 10 years, assuming a linear product development and secure investment. This transitional horizon partially overlaps with early HTAs [49], which are increasingly applied to medical devices to guide research and development, optimize design, and manage risks related to market access and reimbursement [50]. As innovation in MedTech often outpaces regulation, HTA and regulatory bodies require advanced notice to prepare for the assessment of these new, and often disruptive, technologies. This is particularly evident in the case of AI and software, which prompted new regulatory guidance that recognizes software as a medical device [51]. Evidence and weak signal generation during this horizon are incremental and visible, though events remain less predictable than in the imminent horizon.

**Figure 6.**
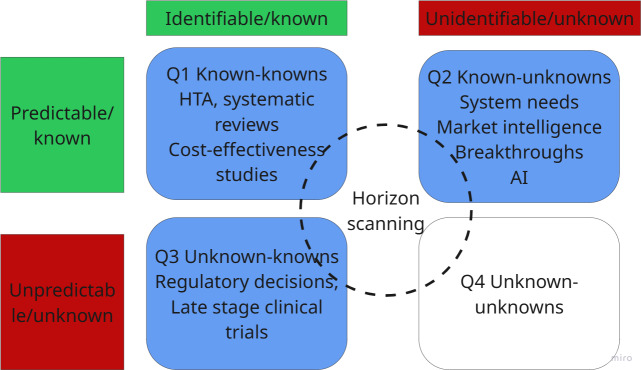
Rumsfeld matrix quadrants in the transitional horizon. AI: artificial intelligence; HTA: health technology assessment.

Uncertainty about the future of a technology’s emergence in this horizon is 2-fold: first, it depends on clinical trial success, market conditions, and secure funding. Second, certainty about the technology’s effectiveness gradually increases as it progresses from pilot to pivotal clinical trials. This horizon involves the known unknowns (Q3) and the known knowns (Q1) from the Rumsfeld matrix [1] but with greater levels of unpredictability. Furthermore, Q2, the known unknowns creep into this horizon as technology “disrupters” or “game changers” whose implications may be overlooked or not fully understood due to biases in the decision-making system [1], for example, with pressures to address immediate issues such as a lack of diagnostic facilities [52,53]. Horizon scanning methods must contend with higher levels of unpredictability, limited visibility of events, and variable timelines for event development (Figure 6).

There are limitations for horizon scanning in the transitional horizon. The use of additional methods in combination with weak signal detection and prioritization, such as scenario analysis or forecasts, can help to deal with high levels of uncertainty by either defining alternative futures or anticipating the timing of an event [1]. Hines and Bishop [9] provide a stepwise approach to dealing with uncertainty in futures planning by engaging the stakeholders in thinking about the challenges and prioritizing the issues or opportunities that may arise, then designing different scenarios of each issue or opportunity, and finally ranking those. Textbox 2 provides the criteria for evaluating these issues or opportunities.

Textbox 2. **Dealing with uncertainty in the transitional horizon: prioritizing issues or opportunities for futures planning.**

- How likely are those issues?
- How much of an impact would the issues have on the future?
- How unprepared are we (the stakeholders) for that future?

In MedTech, key questions include: How likely are these technologies to emerge? How disruptive are these technologies? How prepared is the health care service for the rapid adoption of them? Answers to these questions support preparedness and strategy planning, helping to mitigate some of the uncertainties in the transitional horizon, although many of these uncertainties will resolve or diminish over time.

Dimensional factors also play a role in shaping future technology. The technological dimension is critical, as emerging technologies can accelerate or halt the progress of existing technologies. The economic dimension can also drive or impede development depending on investment flows, while disinvestment or prioritization of resources can be slow to progress. Finally, the political dimension, especially political maneuvers that support or undermine research investment, will directly impact this horizon, particularly for public research [54].

#### Best Fit Methods, Sources, and Tools for Horizon Scanning in the Transitional Horizon

As for the imminent horizon, some of the methods and sources proposed in Table 5 for scanning in the transitional horizon can be used in combination. In this horizon, it becomes evident the widespread use of sources to combat the greater uncertainty posed by the TRLs of the technologies, the lower level of visibility of these weak signals, and the decreased predictability. This mitigation strategy results in an increased number of information sources that need to be searched and results that need to be sifted through to identify weak signals.

**Table 5.** Mapping of methods and sources for the transitional horizon.

Method used	Recommended sources
Rapid evidence synthesis	Published literature
Bibliometric studies	Published literature
Horizon scanning searches	Ongoing clinical trials Market scan Specialist MedTech publications News scanning Funding sites Regulatory agencies
Surveys and questionnaires	Patient and public engagement
Clinical and nonclinical topic expert workshops	Expert consultation
Gray literature searching	Conference proceedings Policies (national or international)
Sentiment analysis	Social media

Consequently, when horizon scanning for technologies in the transitional horizon, we must be aware of the impact of “noise,” understood as “the retrieval of irrelevant signals,” and the risk of “silence” understood as “the failure in retrieving relevant text from a database.” One way to deal with noise is by involving stakeholders and experts early in the process and maintaining that engagement throughout the project. Consulting with experts could alleviate some of the issues generated by noise by the prioritization of weak signals based on their maturity and strength. The issue of silence requires more attention as it could mean different things. On the one hand, it could mean that there is a research gap, funding gap, or innovation gap, but none of these gaps could be fully ascertained without scanning further into the horizon, this is, by scanning the emerging horizon (H3) and contextualizing the findings in the social, political, economic, and technological dimensions (Textbox 1). On the other hand, silence could mean that the search focus is not correct or accurate enough to retrieve the relevant weak signals. This is often an issue faced when practicing horizon scanning for new innovations where the language is not settled or standardized. Bibliometric studies and visualization of similarities techniques can deal with those issues [55].

### Horizon Scanning in the Emerging Horizon (Horizon 3)

#### Overview

In the MIST framework, the emerging horizon is understood as the time when an invention occurs in the shape of a hypothesis that needs testing or is at early experimentation, a patent may be registered and granted, and initial funding is sought for further development of this idea into a prototype. The scope of this horizon is for technologies at an early stage of development (TRLs 1‐3). The time horizon here is very broad and may be highly dependent on factors such as the characteristics of the innovation, the current need for it, previous and accumulative technological advances that may facilitate the realization of this idea in the current time or interest from high-risk investors and venture capitalists [19,56].

This horizon offers a valuable opportunity to explore trends and innovations beyond medical applications, extending across various disciplines. Analyzing these trends can help anticipate the broader impact of emerging technologies. For instance, how quantum computing might affect multiple sectors, including but not limited to health care, or provide context to the gaps identified in the transitional horizon. While this horizon provides abundant data sources, such as patents, preclinical research, and conference abstracts, the complexity of the information and the uncertainty surrounding the weak signals at this early stage demands complementary analytical methods to identify overarching patterns and interconnections across datasets. While weak signal generation in this horizon is rich (registration of intellectual property in patents, presentation of inventions at conferences and grant applications) and visible, events are less predictable; therefore, the level of uncertainty about the future of a technology’s emergence in this horizon is at its greatest point.

**Figure 7.**
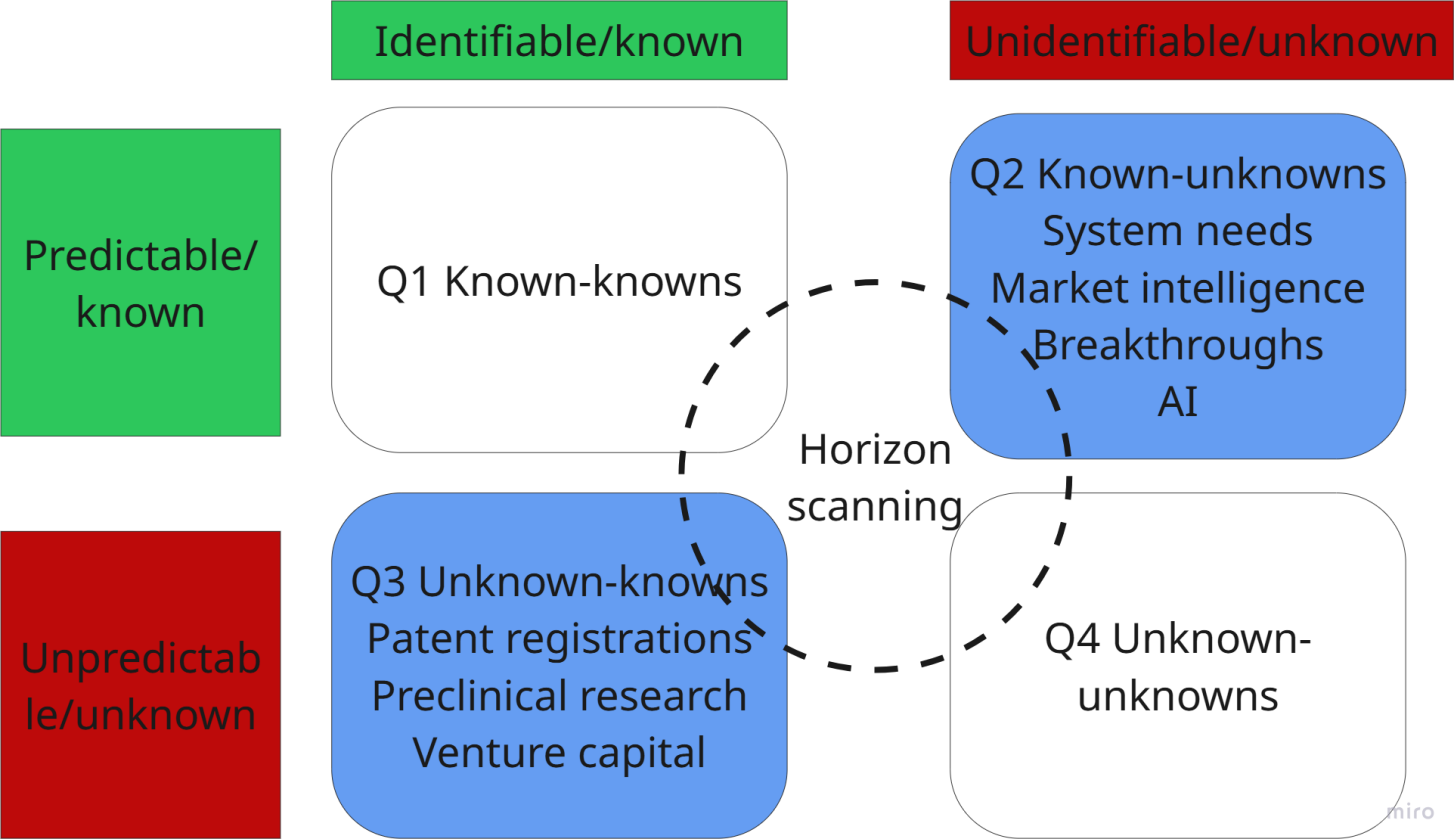
Rumsfeld matrix quadrants in the emerging horizon. AI: artificial intelligence.

In terms of Rumsfeld quadrants (Figure 7), despite the high uncertainty or unpredictability of events unfolding in the emerging horizon or distant future, weak signals are still known (eg, patents, grants, and scientific advances announced at conferences, breaking news about future technology, trends analysis reports, etc). In this horizon, we are mostly operating in the Q2 known-unknowns and Q3 unknown-knowns. We might know that a technological innovation is emerging, but the distant time horizon and unpredictability of whether that technology might progress further and materialize into innovative health care solutions is too high to ascertain. In Q2, we also face surprises in the form of technologies that emerge rather rapidly and almost unexpectedly. This is the case with novel digital health technologies that do not need long research and development pathways.

#### Best Fit Methods, Sources, and Tools for Horizon Scanning in the Emerging Horizon

**Figure 8.**
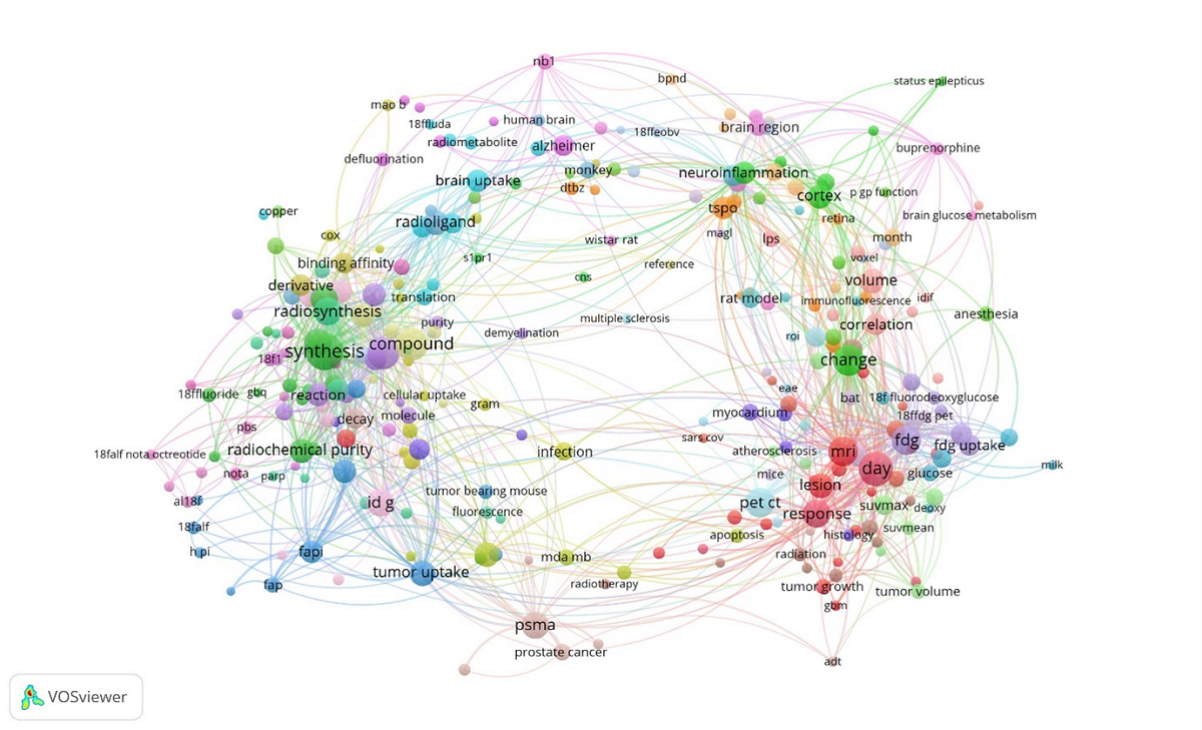
Visualization of similarities between key words in positron emission tomography preclinical research. MRI: magnetic resonance imaging.

In the emerging horizon, the volume of available data is typically greater than at later stages, rendering weak signal detection a process of distinguishing meaningful ones from background noise. An overview of the corresponding approaches, sources, and methods is presented in Table 6. Among these, visualization of term similarities represents a valuable means of mapping and interrogating research landscapes. We used this approach for the visualization of co-occurring terms extracted from Embase (Ovid) records of preclinical studies on fluorine-18 and positron emission tomography [53]. This analysis sought to characterize the early innovation landscape of fluorine-18 applications to provide anticipatory intelligence for decision-makers. Within the network representation (Figure 8), the size of each node reflects term frequency, with prominent terms such as synthesis, radiosynthesis, tumor uptake, prostate-specific membrane antigen, lesion, and response denoting areas of concentrated research activity. By contrast, smaller, more peripheral nodes, such as neuroinflammation linked to status epilepticus, cortex, and brain region, point to emerging or niche areas of investigation, including the application of fluorine-18 positron emission tomography for monitoring neuroinflammation.

**Table 6.** Mapping of methods and sources for the emerging horizon.

Method used	Recommended sources
Horizon scanning searches	Market scan Specialist MedTech publications News scanning Funding sites
Sentiment analysis	Social media
Horizon scanning searches; text mining; cluster visualization techniques	Patent databases (European and worldwide)
Bibliometric and scientometric methods: visualization of term similarities, cocitation analysis, cluster analysis	Bibliographic databases
Workshop or survey	Experts in the research field

Overall, similarity-based visualization constituted an effective method for uncovering research clusters and nascent applications within the radionuclide field. Although this technique cannot be expected to retrieve all possible data, it can highlight early weak signals and generate hypotheses for more detailed exploration. Crucially, domain expertise remains indispensable for interpreting network structures and associations, thereby ensuring the accurate identification of weak signals and their relevance within the broader research context [53].

Patent data is also rich in this horizon and presents a great body of data for trend analysis due to patent data availability across jurisdictions, making patent analysis studies a suitable method for identifying which patents are on the rise in different regions and study trends [57].

## Discussion

### Principal Findings

Human existence is defined by the quest for knowledge and the humble acknowledgment that there is much that we do not know. While horizon scanning is a method designed to detect weak signals, the “unknown unknowns” will not produce signals; should there be any, then these automatically by definition will move into the realm of the “knowns” following the premise “if we can think of it, then it exists and then is not unknown” (Garvey et al, 2022) [1]. Horizon scanning does not belong to the exploration of the “unknown unknowns,” and probably nothing belongs to this quadrant (Q4) of the Rumsfeld matrix other than postevent justifications for why we did not know or realize that something would happen [1]. This is essentially the field of hindsight, and one not included in the scope of this methodological framework.

Within this context, the Rumsfeld matrix and the Three Horizons model are used in MIST as established foresight tools rather than as new methodological contributions in their own right. The novelty of MIST lies not in proposing these models for the first time, but in combining and operationalizing them within a MedTech-specific horizon scanning framework. In particular, MIST links these broader conceptual tools to TRLs, regulatory pathways, source selection, and stakeholder decision needs, thereby translating general foresight theory into a structured and practical methodology for MedTech horizon scanning.

The framework responds to a very specific need for horizon scanning methods in a very specific field. The high specificity of the method is geared toward guiding the practice of horizon scanning to solve uncertainty in this field and help decision-making needs in MedTech, as opposed to more generalist frameworks [11,12,17,58] that are designed to broadly guide foresight practice, of which horizon scanning may be a component. Furthermore, the development of this framework is underpinned by extensive expert engagement and methodological practice. Input was gathered from a diverse group of specialists spanning horizon scanning, HTA, regulatory science, futures studies, and MedTech innovation, ensuring that the framework reflects a broad range of practical and conceptual perspectives. The multidisciplinary expertise of the project’s team further strengthens the robustness of the framework, particularly given the IO’s long-standing experience in horizon scanning, information retrieval, and evidence synthesis for health and care technologies.

Horizon scanning in the imminent horizon is also valuable in its own right because it supports early awareness and preparedness for HTA agencies and related decision-makers. Even when technologies are close to market entry, structured scanning remains important to identify those likely to require assessment, anticipate evidential and implementation challenges, and support timely planning for appraisal, adoption, or service response. In this sense, the imminent horizon is not outside the scope of horizon scanning but represents a critical stage at which anticipatory intelligence directly informs HTA and policy processes. This near-term anticipatory function is particularly relevant across jurisdictions, even though the specific regulatory and decision-making structures may differ. While the methodological core of MIST is jurisdiction-agnostic (universal functions such as early identification, filtering, characterization, prioritization, and iterative monitoring), some operational anchors may be configurable to other jurisdictions, equivalent regulatory and policy anchors (eg, FDA, Pharmaceuticals and Medical Devices Agency, National Medical Products Administration, and Canadian Agency for Drugs and Technologies in Health) without altering the framework’s underlying logic. MIST should therefore be understood as modular: methodologically universal but locally instantiated through jurisdiction-specific regulatory and system contexts.

Our work has some limitations. Our framework has not been formally validated; therefore, we foresee the need to revise some of the methods and sources proposed here in the near future. Many components are interdependent in this framework; one change may affect others, and we recommend considering MIST as the first version of a living framework that would need updating regularly to incorporate the latest methodological or regulatory advances. While equity and ethical considerations are critical in the adoption and assessment of MedTech innovations, these dimensions are not explicitly incorporated in the current iteration of the MIST framework. The development of MIST has focused on establishing a robust, transparent, and systematic methodological foundation for horizon scanning in MedTech, addressing challenges such as rapid product lifecycles, weak signal detection, regulatory variability, and stakeholder integration. Parallel work with key stakeholders is ongoing to develop methodologies for embedding equity and ethical principles as a cross-cutting layer across horizons into MedTech decision-making processes [59,60]. The insights gained from these initiatives will inform the next iteration of MIST, ensuring that future versions of the framework comprehensively integrate equity, fairness, and ethical considerations alongside methodological rigor.

### Conclusion

Horizon scanning for MedTech innovation faces multiple methodological, practical, and conceptual challenges. MedTech products are highly heterogeneous, encompassing medical devices, in vitro diagnostics, and digital health solutions, and often undergo rapid, iterative development with short lifecycles. Continuous technological advances in AI, sensors, materials, and software can quickly render previous versions obsolete, while fragmented data sources, weak and uncertain signals, and diverse stakeholder perspectives complicate robust identification and prioritization of emerging technologies [16]. Regulatory complexity, including differing regional requirements and evolving standards, adds further uncertainty, and inconsistent terminology coupled with the absence of standardized reporting frameworks limits transparency, comparability, and reproducibility [8].

Although several horizon scanning frameworks exist, no framework has been published to guide the practice of horizon scanning in the context of emerging and innovative health care technologies and, more specifically, in MedTech. To address these challenges, we have developed MIST, a systematic framework that provides structured, unbiased, and transparent guidance for horizon scanning in MedTech innovation. MIST is designed to support national policy and decision-makers in making timely, evidence-informed decisions regarding the adoption of new technologies, accelerate implementation into clinical practice, and identify unmet research and investment priorities, while addressing the specific methodological and operational complexities of MedTech horizon scanning. At this stage, MIST should be understood as a conceptually derived and practice-informed framework; quantitative evaluation of its components and application across case studies will form part of future validation work.

## Abbreviations

AI: artificial intelligence
CE: Conformité Européenne
FDA: US Food and Drug Administration
HTA: health technology assessment
IO: Innovation Observatory
MDR: Medical Devices Regulation
MIST: MedTech Innovation Scanning Techniques
TRL: technology readiness level
UKCA: UK conformity assessment
